# How I do it: the trans-laminar, facet-joint sparing minimal invasive approach for ventral dural repair in spontaneous intracranial hypotension—a 2-dimensional operative video

**DOI:** 10.1007/s00701-021-04987-w

**Published:** 2021-09-04

**Authors:** Marco V. Corniola, Torstein R. Meling

**Affiliations:** 1 Neurosurgery Division, Department of Clinical Neurosciences, Geneva University Hospitals, Geneva, Switzerland; 2 Faculty of Medicine, Geneva University, Geneva, Switzerland; 3 Neurosurgery Division, Department of Neurosciences, Rennes University Hospital, Rennes, France

**Keywords:** Minimally invasive surgery, Spontaneous idiopathic hypotension, Microsurgery, Subdural hematoma

## Abstract

**Background:**

We describe the minimally invasive, facet-sparing postero-lateral approach to the thoracic spine for a ventral dural repair in a patient with intracranial hypotension secondary to a spontaneous dural breach.

**Methods:**

We performed a minimally invasive approach using a short paramedian posterior skin incision followed by a 10 × 10 mm targeted trans-laminar approach, to achieve a microsurgical repair of a symptomatic ventral dural defect causing severe disability.

**Conclusion:**

The facet-sparing postero-lateral approach is safe and effective in the surgical management of thoracic dural tears, even in the most anterior ones, and avoids the traditional costotransversectomy.

**Supplementary Information:**

The online version contains supplementary material available at 10.1007/s00701-021-04987-w.

## Introduction

Spontaneous intracranial hypotension is a rare disease with an estimated incidence of 5/100,000, primarily affecting patients in their fourth and fifth decade [2]. It is caused by a spinal dural cerebrospinal fluid (CSF) leak, which mostly occurs along the cervico-thoracic spine [1], and the dural tear is often inflicted by a degenerative calcified discogenic microspur. In the case of a significant CSF leakage, brain sagging puts meninges, veins, and cranial nerves under tension and may cause symptoms like orthostatic headache [3]. Whenever SIH is suspected, dynamic CT myelography is the gold standard imaging to precisely localize the CSF leak [2, 4] and the associated microspur. We present the case of a 40-year-old healthy female with symptomatic SIH. The diagnosis and the technical aspects are thoroughly presented and the most recent literature on the topic is discussed.

## Relevant surgical anatomy

**Fig. 1:**
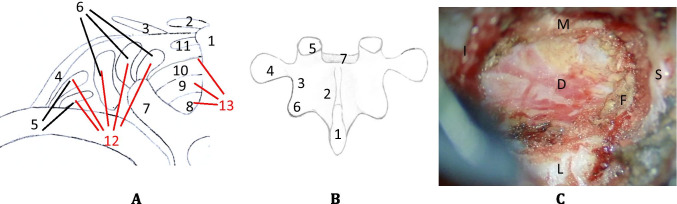
**A** (1) Spinous process; (2) M. trapezius; (3) M. latissimus dorsi; (4) M. serratus posterior; (5) M. iliocostalis; (6) M. longissimus; (7) Transverse process; (8) Rotatores muscles; (9) M. multifidus; (10) M. semispinalis; (11) M. spinalis; (12 = 5 and 6): Erector spinae muscles; (13 = 8–10): Transversospinalis muscles. **B** (1) Spinous process; (2) Lamina; (3) Isthmus (pars interarticularis); (4) Transverse process; (5) Superior articular process; (6) Inferior articular process; (7) Spinal canal. **C** (M) Medial; (L) Lateral; (S) Superior; (I) Inferior: (D) Dura; (F) Flavum (sub-totally resected)

The macroscopic and microscopic surgical anatomies of the surgical approach are shown in Fig. 1. The skin incision is paramedian, allowing a larger working angle and a higher range of motion for instruments during the surgical procedure. Attention should be paid to keep the surgical field as dry as possible, avoiding unnecessary bleeding during surgical exposure. A subperiosteal dissection of the muscles should be carefully performed. However, the full exposure of the entire zygapophyseal articulation is unnecessary, and the joint capsule must not be violated.

During the intradural step, attention must be paid not to push against the spinal cord while exposing the ventral dural defect. To facilitate spinal cord mobility, the denticulate ligament may be cut. In our case, this was not necessary.

## Description of the technique

**Fig. 2:**
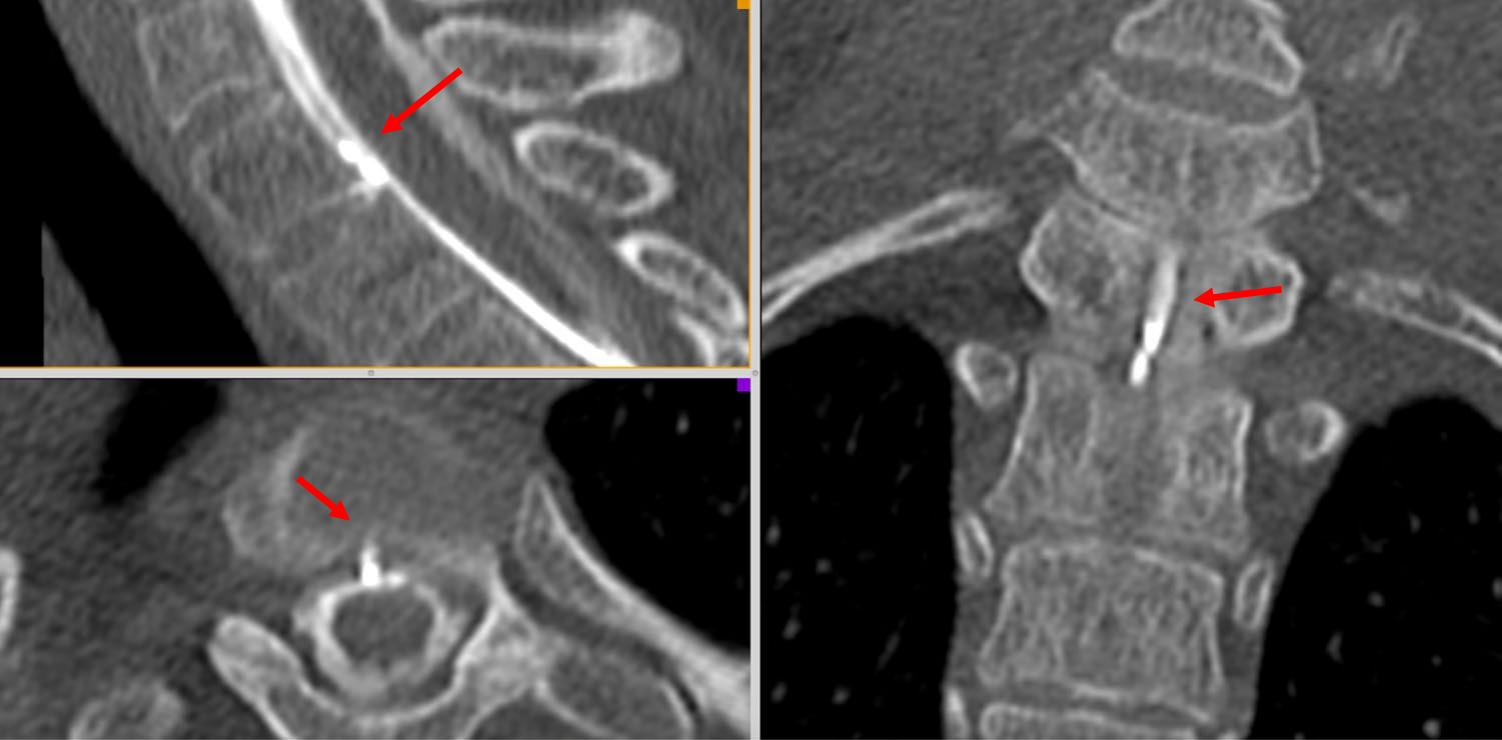
Pre-operative myelo-CT scan showing the ventral dural leak

After the identification of the CSF leak on a myelo-CT (Fig. 2), the surgical indication was retained. The patient was positioned prone with the head maintained in a neutral position with a Mayfield head clamp. Cefazolin 2 g was given i.v. as prophylactic antibiotic. Motor and somato-sensory evoked potentials were used. Precise localization of the level was verified using fluoroscopy, and the surgical site disinfected by Betadine followed by sterile draping.

A straight, slightly paramedian 3-cm-long skin incision was performed (right-sided), centered at the level of the CSF fistula. The lumbodorsal fascia was incised with the monopolar, and the ipsilateral paravertebral muscles were dissected. A careful and thorough hemostasis was achieved, followed by the installation of a Fehling retractor.

The bone plane was exposed using the monopolar. Thereafter, the interlaminar window was enlarged to 10 × 10 mm using a 4-mm sharp matchstick drillbit. Bone wax was used to secure the hemostasis. The flavum was resected using a Kerrison punch, exposing the underlying dura. Throughout the procedure, immaculate epidural hemostasis was secured, allowing uninterrupted and pristine microsurgery. This is paramount, especially after the dural opening, in order to avoid blood in the subarachnoid space, carrying the risk of secondary inflammatory arachnoiditis.

**Fig. 3:**
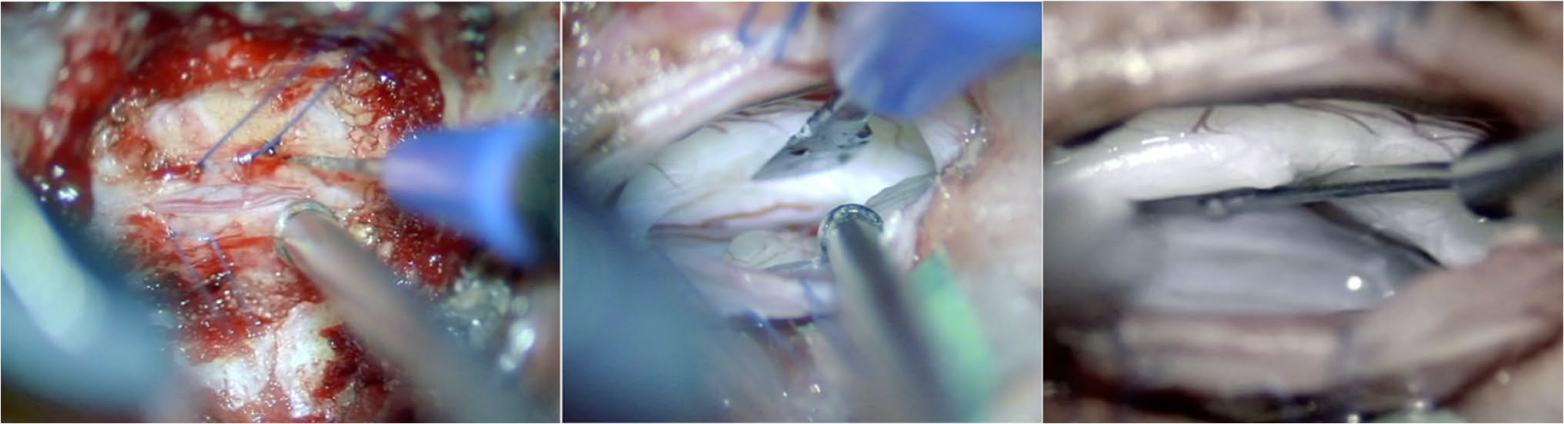
The dural incision was performed dorso-laterally to allow the surgeon to work under the spinal cord with very minimal retraction

**Fig. 4:**
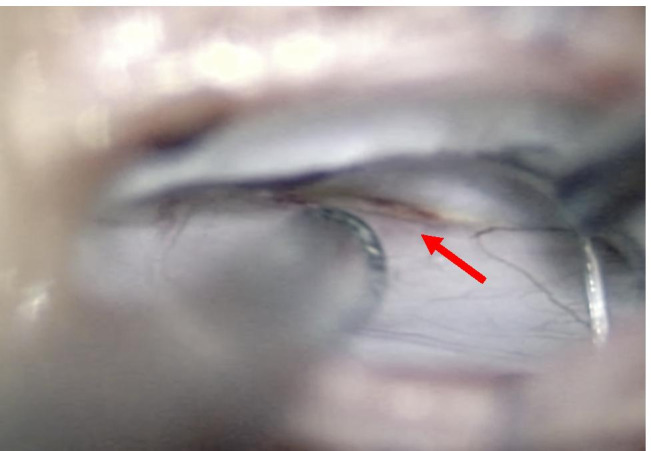
Intra-operative microscopic view of the ventral dural defect

Prolene 5–0 stitches were used to suspend the dura, which had been incised longitudinally. The dural incision was performed dorso-laterally to allow the surgeon to work under the spinal cord, with minimal retraction (Fig. 3). CSF was released and the dural suspensions completed. A careful inspection of the ventral dura using a microdissector was undertaken with special attention to the ventral spinal cord. A thorough circumferential inspection of the ventral dural defect was achieved (Fig. 4), and the underlying bone spur could be removed using a microcurette, until no more bone irregularities were palpated.

**Fig. 5:**
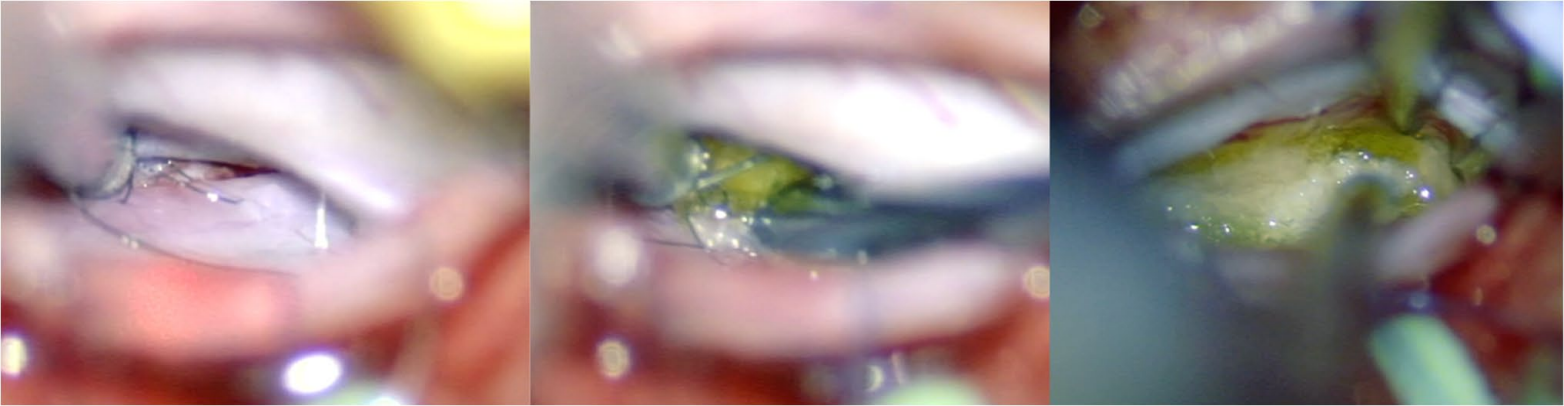
To repair the fistula, the dural edges are approximated with a Prolene 9–0 running suture followed by a TachoSil® sandwich technique. The suture loops are initially kept lose to allow for the passing of a TachoSil® sponge extradurally as an outlay. The suture is then tightened and tied, whereafter a supplementary TachoSil® sponge is placed intradurally as an inlay. Note that the sticky (yellow) faces are placed against the dura

The dura surrounding the defect was dissected from the bone, so as to make room for a piece of TachoSil®. Running stitches with Prolene 9–0 were placed at the dural edges and the dura repaired using a TachoSil® “sandwich technique” (Fig. 5). In details, the first TachoSil® sponge was placed extradurally, facing the dural defect, and the Prolene suture was tightened to reduce the dural defect. A gentle pressure was applied in order to secure a good adhesion between the dura and the TachoSil. A second piece of TachoSil® was then placed intradurally, with the sticky side facing the dural defect and the first sponge and gentle pressure was applied to the construct for about 1 min. The sizes of these pieces were about twice the size of the dural defect so as to ensure good adhesion between the TachoSil® and the surrounding dura.

A Prolene 6–0 running suture was used to close the postero-lateral dural opening. A piece of TachoSil was added extradurally, followed by a regular myofascial and subcutaneous closure using a Monocryl 2–0 running suture and a skin closure using a Prolene 4–0 running suture.

**Fig. 6:**
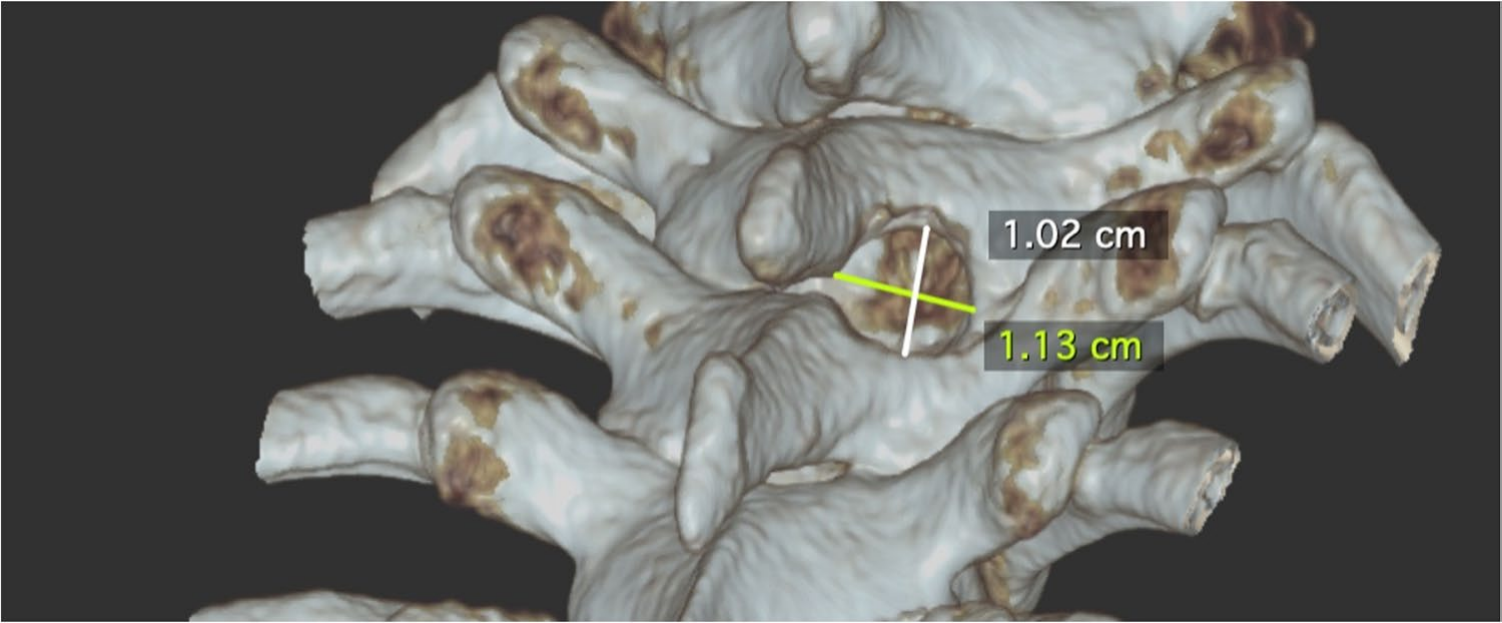
Postoperative 3D bone reconstruction of the minimal invasive trans-laminar, facet-sparing approach to the spinal canal, with a maximal diameter of 11 mm

**Fig. 7:**
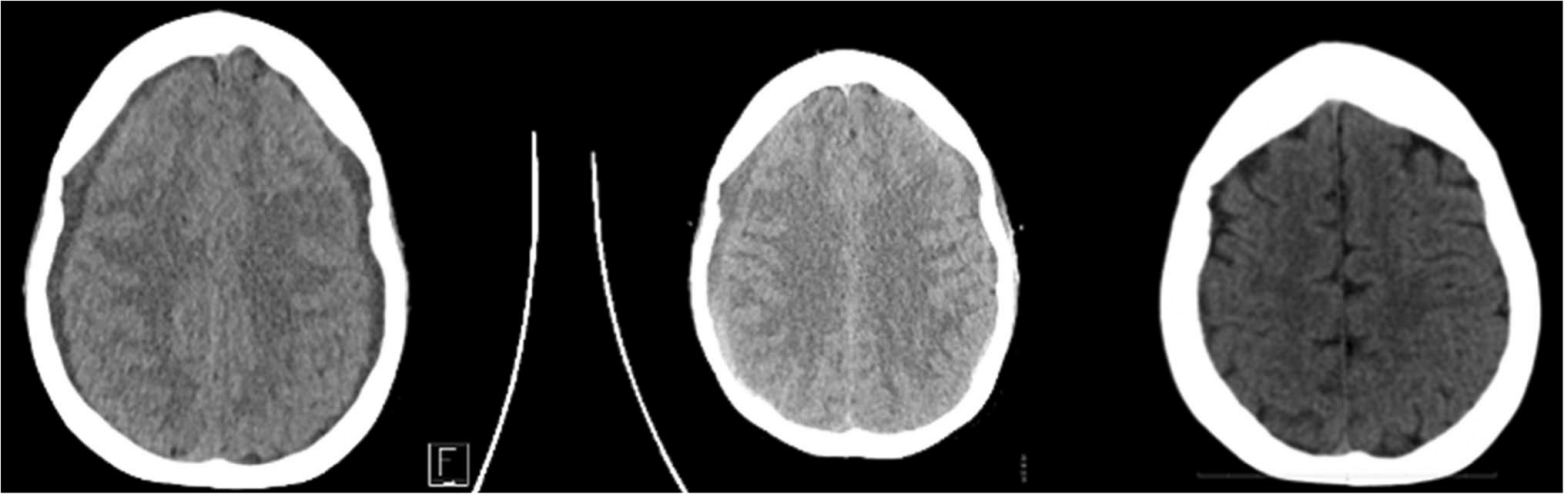
Postoperative CT scan at one, 2 and 3 months (from left to right) showing the complete resolution of the subdural hematomas

After surgery, the patient was kept in bed for 48 h before gradual mobilization (due to her significant subdural hygromas). The postoperative course was free of any complication and the symptoms completely resolved. A 3D reconstruction of the postoperative CT scan shows the minimal invasive interlaminar bone window (Fig. 6). Postoperative CT scans show the complete resolution of the subdural hematomas (Fig. 7).

## Indications

The postero-lateral trans-laminar, facet-sparing approach is suitable for lesions of the spinal canal and allows a safe, straightforward, and effective approach to the ventral dura, without the need to resect parts of the zygapophyseal joint or rib to access to the anterior part of the spinal canal. This approach is a valid alternative to the potentially destabilizing costotransversectomy, traditionally used when it comes to access to the most ventral aspect of the thoracic dura.

## Limitations

While the surgical management of SIH may appear technically demanding, it is not a major obstacle to cure the patient. In our opinion, the accurate diagnosis of SIH with proper imaging and accurate visualization of the exact level of the fistula is of paramount importance. However, the surgery should be performed by a trained microneurosurgeon, mastering advanced microsurgical techniques and sutures. The face-sparing approach is not suitable in the case of large anterior lesions, such as spinal meningiomas. Patients with zygapophyseal hypertrophy may not be candidates to the minimally invasive, facet-sparing approach: a careful pre-operative radiological assessment should be undertaken to ensure safety and feasibility for a specific patient.

## How to avoid complications

Careful patient positioning and radiological identification of the correct level are paramount. Spinal navigation can be used to ensure that an adequate angle of view is obtained. During muscle dissection, keep the surgical field dry and clean, since blood contamination can prevent optimal visualization of the critical structures, such as the dura (and dural tear) and the spinal cord. Furthermore, arachnoidal contamination with blood can cause subsequent inflammatory arachnoiditis and adhesions, which are very difficult to cure, even with adequate management. Therefore, immaculate epidural hemostasis is paramount for allowing uninterrupted pristine microsurgery, especially after the dural opening.

Watertight closure of the dural tear using running sutures with inlay and outlay reinforcement is also important, since surgical failure requires re-operation.

In case of bleeding of the spinal cord, electrocautery must never be used. Instead, gentle compression with cottonoids along with smooth rinsing is sufficient. Whenever blood contamination occurs during the intradural phase, a thorough low-pressure rinsing must be performed. Do not use the drill to resect the bone spurs, since the spinal cord does not tolerate even minimal heating.

## Specific information for the patient

Patients should be warned about general risks of the surgery, e.g., hematoma, postoperative infection, CSF leaks, pseudo-meningocele, and failure-to-cure. It is of outmost importance that patients understand that while the symptoms are mostly cranial, the problem comes from the spinal canal. Patients should be warned that the surgery can be converted to a standard costotransversectomy in case of failure to achieve adequate exposure, increasing the risk of postoperative pain and secondary destabilization of the spine.

## 10 key point summary

1. SIH secondary to a spontaneous CSF leak is rare disease, but can cause severe symptoms.
2. Myelo-CT is the best imaging modality to identify and localize precisely CSF leaks in case of SIH.
3. Microsurgical closure is the first-line therapy of SIH. The trans-laminar, facet-joint sparing technique is much less invasive than the standard costotransversectomy, but requires advanced microsurgical skills and should be performed by a senior microneurosurgeon.
4. Careful perioperative localization using fluoroscopy should be performed in order to ensure the minimal invasive aspect of the procedure.
5. Avoid unnecessary bleeding during the extradural approach, since arachnoidal contamination with blood may result in postoperative adhesions.
6. Use the so-called “sandwich technique” with and inlay and outlay in addition with the suture of the dural tear (products with fibrin-covered surfaces may be preferred).
7. If necessary, sectioning of the denticulate ligament can increase the surgical view.
8. When bone spurs must be removed, do not use the high-speed drill in the vicinity of the spinal cord.
9. Whenever bleeding of the spinal cord occurs, apply gentle pressure using cottonoids; do not use electrocautery.
10. When achieved successfully, the surgical closure of the dural tear results in immediate postoperative pain relief.

## Conclusion

The minimally invasive trans-laminar, facet-joint sparing approach to repair anterior thoracic CSF fistulas is safe and effective, allowing for speedy recovery of symptomatic patients. A postero-lateral dural incision avoids unnecessary and harmful spinal cord mobilization. A watertight closure using a combination of Prolene 9–0 suture and a TachoSil® “sandwich technique” secures the dural repair and enables early mobilization.

## Supplementary Information

Below is the link to the electronic supplementary material.

Supplementary file1 (MP4 321891 KB)
